# 4-Hydroxy-7-methyl-3-phenylcoumarin Suppresses Aflatoxin Biosynthesis via Downregulation of *aflK* Expressing Versicolorin B Synthase in *Aspergillus flavus*

**DOI:** 10.3390/molecules22050712

**Published:** 2017-04-29

**Authors:** Young-Sun Moon, Leesun Kim, Hyang Sook Chun, Sung-Eun Lee

**Affiliations:** 1 School of Applied Biosciences, Kyungpook National University, Daegu 41566, Korea; space92@knu.ac.kr (Y.-S.M.); twosuns.kim@gmail.com (L.K.); 2 Advanced Food Safety Research Group, BK21 Plus, School of Food Science and Technology, Chung-Ang University, Anseong 17546, Korea; hschun@cau.ac.kr

**Keywords:** 4-hydroxy-7-methyl-3-phenyl coumarin, 2,3-dihydrobenzofuran, aflatoxin production, *Aspergillus flavus*, reverse transcription polymerase chain reaction

## Abstract

Naturally occurring coumarins possess antibacterial and antifungal properties. In this study, these natural and synthetic coumarins were used to evaluate their antifungal activities against *Aspergillus flavus*, which produces aflatoxins. In addition to control antifungal activities, antiaflatoxigenic properties were also determined using a high-performance liquid chromatography in conjunction with fluorescence detection. In this study, 38 compounds tested and 4-hydroxy-7-methyl-3-phenyl coumarin showed potent antifungal and antiaflatoxigenic activities against *A. flavus*. Inhibitory mode of antiaflatoxigenic action by 4-hydroxy-7-methyl-3-phenyl coumarin was based on the downregulation of *aflD*, *aflK*, *aflQ*, and *aflR* in aflatoxin biosynthesis. In the cases of coumarins, antifungal and aflatoxigenic activities are highly related to the lack of diene moieties in the structures. In structurally related compounds, 2,3-dihydrobenzofuran exhibited antifungal and antiaflatoxigenic activities against *A. flavus*. The inhibitory mode of antiaflatoxigenic action by 2,3-dihydrobenzofuran was based on the inhibition of the transcription factor (*aflS*) in the aflatoxin biosynthesis pathway. These potent inhibitions of 2,3-dihydrobenzofuran and 4-hydroxy-7-methyl-3-phenyl coumarin on the *Aspergillus* growth and production of aflatoxins contribute to the development of new controlling agents to mitigate aflatoxin contamination.

## 1. Introduction

Aflatoxins including AFB1, AFB2, AFG1, and AFG2 are mycotoxins produced by *Aspergillus flavus* and *A. parasiticus,* with potent carcinogenic activity, especially on human liver [1]. Aflatoxins can be accumulated in humans and livestock through diet of aflatoxin-contaminated foods and feed [2,3]. Outbreaks of aflatoxicosis are notably dependent on the crop species and seasonal changes of a given region [4,5]. Additionally, they are also related to poor agricultural practices [6]. Therefore, alternative agricultural practices may be needed to develop mitigating aflatoxin contamination in crops.

Chemical control of fungal growth and aflatoxin production has been successfully documented using propionic acid in unshelled peanuts on the laboratory scale [7]. In the crop field, usage of fungicides is critical to control fungal growth and mycotoxins with good efficacy [8]. Recently, phytopathogens develop resistance to various fungicides [9,10]. With this reason, alternatives for controlling *Aspergillus* infection and aflatoxin contamination are highly needed, and natural products could be considered as candidate compounds.

Coumarins are naturally occurring compounds produced after cyclization of cinnamic acid via formation of phenylpropanoids through the shikimic acid pathway [11]. Recently, plant-specific coumarins such as umbelliferone and scopoletin have been produced in *E. coli* due to their various applications after enzyme-engineered conversion with or without inexpensive precursors, 4-coumaric acid and ferulic acid [12]. Coumarins possess antibacterial activity against *Ralstonia solanacearum* [13], antimicrobial activity against *Staphylococcus aureus* [14], and antifungal activities against clinically important fungal pathogens [15]. Coumarins with antioxidant activities inhibit aflatoxin formation because aflatoxin formation occurs when fungal species are subject to oxidative stress [16,17,18]. In addition to this finding, a structure–activity relationship (SAR) study of 24 coumarin derivatives showed that *O*-substitutions seem to be essential for antifungal activity against *A. flavus* and *A. fumigatus* [19]. However, the authors did not study the relationship between the structure of coumarins and the antiaflatoxigenic activity generated by *A. flavus*. In the shikimic pathway, indole is generated and its derivative has shown antifungal activity [20].

In our previous studies, we have found that methylenedioxy-containing natural and synthetic compounds possessed antifungal and antiaflatoxigenic properties against *A. flavus* [21]. In this study, 1-(2-methylpiperidin-1-yl)-3-phenylprop-2-en-1-one showed potent antifungal and antiaflatoxigenic activities against *A. flavus* among the tested 22 compounds, and its mode of inhibitory action on aflatoxin production was caused by inhibition on the expression of some genes involved in aflatoxin biosynthesis such as *aflD*, *aflK*, *aflQ*, *aflR*, and *aflS* [21]. Other reports have shown that natural products are good candidates as preservatives to suppress aflatoxin contamination in cereals and feedstuffs [22,23,24].

In the present study, 26 coumarins were assessed to determine their antifungal activities against *A. flavus* and the inhibitory effects on aflatoxin production. The mode of inhibitory action on the aflatoxin production was disclosed using real-time PCR. Further studies for antifungal and antiaflatoxigenic activities were undertaken using structurally closed compounds including 2,3-dihydrobenzofuran, indole, 1-methyl indole, 2-methyl indole, 3-methyl indole, and 2-phenyl indole to coumarins for understating relationships between the structure of tested compounds and antifungal and antiaflatoxigenic activities. These antifungal and antiaflatoxigenic substances can be used for controlling *A. flavus* and reducing aflatoxin contamination in agricultural fields before harvest given their ability to decrease aflatoxin production. 

## 2. Results and Discussion

**Figure 1:**
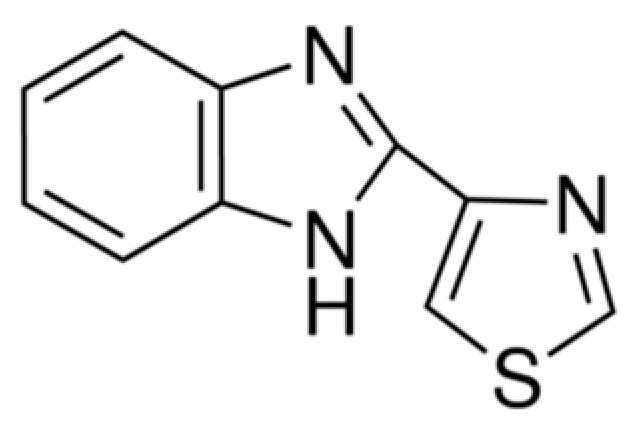
Molecular structure of thiabendazole used as a positive control in this study.

The inhibitory effects of the 32 tested compounds on *A. flavus* growth and aflatoxin production were measured and the results are expressed in Table 1. A currently used fungicide thiabendzole (Figure 1) was used as a positive control and all data were calculated on the basis of the inhibition rate (%) in comparison to the solvent-treated controls [21]. Among the tested coumarins, five compounds showed antifungal activities against *A. flavus*. Among them, 4-hydroxy-7-methoxy-3-phenylcoumarin (**1**) and 4-hydroxy-6,7-dimethylcoumarin (**2**) exhibited about 50% inhibition on the fungal growth at the concentration of 100 μg/mL. However, this inhibitory effect of (**2**) disappeared after exposure to 10-fold diluted concentration (Table 1). 6,7-Dimethoxycoumarin (**3**) also possessed inhibitory effects on the fungal growth at concentrations of 1000 μg/mL. However, this inhibition was no longer evident following treatment with a 10-fold lower concentration of the compound. At the concentration of 10 μg/mL, there was no inhibitory effect by Compound **3** (Table 1).

4-(Bromomethyl)-6,7-dimethoxycoumarin (**4**) and 2,3-dihydrobenzofuran (**5**) showed potent antifungal activities at concentrations of 1000 μg/mL. At the concentration of 100 μg/mL, Compound **4** reduced 63% of fungal growth and Compound **5** completely lost its inhibitory effect. The inhibitory effect of **4** on the *A. flavus* growth was not found at the concentration of 10 μg/mL (Table 1).

The inhibition of aflatoxin production by coumarins was remarkable (Table 2). At a concentration of 10 μg/mL, **1** and **2** showed almost complete inhibition of aflatoxin production. This inhibition was no longer evident following treatment with a 10-fold lower concentration of the compound (Table 2). Compound **1** exhibited potent inhibitory effects on AFB_1_ and AFB_2_ production until treatment with the compound at a concentration of 1 μg/mL. This compound significantly enhanced production of AFG_1_ after the treatment of 100 μg/mL (Table 2). Compound **2** exhibited potent inhibitory effects on AFB_1_ and AFB_2_ production until treatment with the compound at a concentration of 10 μg/mL. At a concentration of 1 μg/mL, the antiaflatoxigenic activity of **5** was observed to be 40% inhibition of AFB_1_ production (Table 2).

The fungicidal and bactericidal activities of coumarin and coumaric acid have been tested against *A. flavus* and *o*-coumaric acid inhibited aflatoxin production, but no correlation with fungal growth was found [25]. In that report, the authors found the complete inhibition of coumarin on fungal growth against *A. flavus* at the level of 10 mmol/L, equivalent to about 1460 μg/mL [25]. This is similar to our result, where most coumarins possessed potent antifungal activities at a concentration of 1000 μg/mL (Table 1).

Coumarins showed similar inhibitory patterns on aflatoxin production, enhancing the production of AFG_1_ (Table 2). It is likely that coumarins inhibit the production of AFB_1_, AFB_2_, and AFG_2_, but promote that of AFG_1_. Various coumarins generally use similar target enzymes involved in the aflatoxin biosynthesis pathway to inhibit aflatoxin production; however, the pathway for production of AFG_1_ escapes inhibition.

Other report using three natural furanocoumarins such as xanthotoxin, bergapten, and psoralen exhibited potent antiaflatoxigenic activities at the 5 mM concentration, but not for antifungal activities due to only 20% inhibition on fungal growth [26]. Holmes et al. [27] reviewed diverse biomolecules for their inhibitory effects on aflatoxin biosynthesis. Coumarins containing bergapten, *p*-coumaric acid, psoralene, and xanthotoxin possessed strong antiaflatoxigenic activities with IC_50_ values below 0.1 mM. Among the known biomolecules, α-ionone (IC_50_ value, 0.4 μM) was the strongest compound to suppress aflatoxin production [28].

In a recent report, authors demonstrated that AFG2 production in *A. flavus* was enhanced after exposure to piperonal, a methylenedioxy-containing compound [24]. In the same report, methyleugenol, a monoterpene, suppressed AFB1 and AFB2 generation, while AFG1 production increased in *A. flavus*. Taken together, chemicals can change the AFB biosynthesis pathway. In contrast, Compound **5** exhibited potent antifungal and antiaflatoxigenic activity in comparison to the positive control, thiabendazole. At concentrations of 1 μg/mL, thiabendazole showed more than 80% inhibition, and Compound **5** showed about 40% inhibition of AFB1 production. These findings are notable, as Compound **5** is a natural product that has potential for use as a major compound in the synthesis of new antiaflatoxigenic compounds.

**Figure 2:**
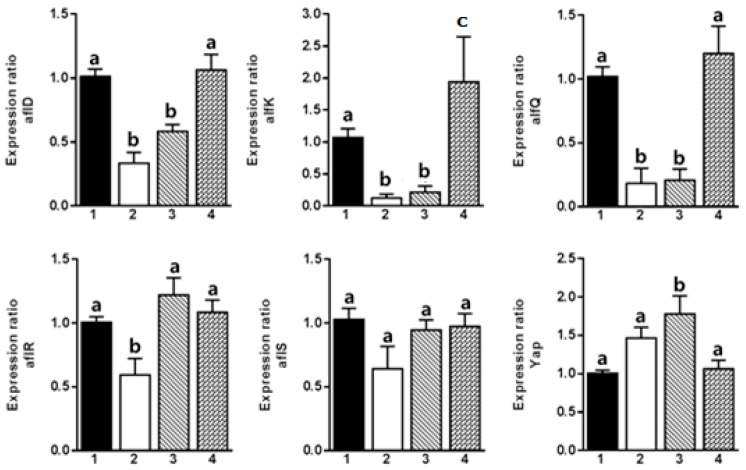
RT-PCR results of aflatoxin biosynthesis using six genes (*aflD*, *aflK*, *aflQ*, *aflR*, *aflS*, and *yap*) regulated by 4-hydroxy-7-methyl-3-phenyl coumarin (**1**). 1: control; 2: 1000 μg/mL of **1**; 3: 100 μg/mL of **1**; 4: 10 μg/mL of **1**). Different letters indicate statistically significant differences between experimental groups analyzed by a Student’s *t*-test (*p* < 0.05).

**Figure 3:**
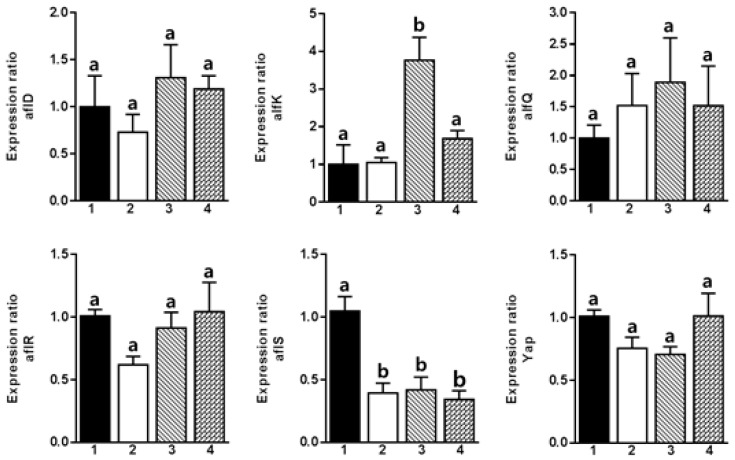
RT-PCR results of aflatoxin biosynthesis using six genes (*aflD*, *aflK*, *aflQ*, *aflR*, *aflS*, and *yap*) regulated by 2,3-dihydrobenzofuran (**5**). 1: control; 2: 1000 μg/mL of **5**; 3: 100 μg/mL of **5**; 4: 10 μg/mL of **5**). Different letters indicate statistically significant differences between experimental groups analyzed by a Student’s *t*-test (*p* < 0.05).

RT-PCR results showed that Compound **1** downregulated *aflR*, *aflD*, *aflK*, and *aflQ*, thereby inhibiting the expression of several genes involved directly in the biosynthesis of aflatoxins (Figure 2). However, Compound **5** suppressed the expression of *aflS* only, which plays an important role in the transcription of genes involved in the biosynthesis of aflatoxins (Figure 3). The gene *aflD* expresses reductase mediating norsolorinic acid (NOR) to averantin (AVN), while *aflK* expresses versicolorin B (VERB) synthase to form VERB from versiconal (VAL). *aflQ* is responsible for oxidoreductase expression, which mediates the formation of aflatoxins. 

The expression of *aflO* and *aflQ* is positively correlated to the production of AFB1 in *A. flavus* [29]. This finding is similar to the results of **1**, which suppressed *aflQ* expression. Recent reports support that the expression pattern of *aflS* gene is related to aflatoxin B1 in *A. flavus* [30,31]. However, the expression of *aflS* varied with aflatoxin-producing ability [32]. Therefore, this finding is related to the non-changeable expression pattern on *aflS* by (**1**), even it possessed inhibitory effects on expression of some genes involved in aflatoxin biosynthesis.

Conclusively, Compounds **1** and **5** among 32 tested compounds exhibited their potent inhibitory effects on *A. flavus* growth and aflatoxin production. The inhibitory effect of Compounds **1** and **5** on fungal growth was observed at a concentration of 100 μg/mL. Compound **1** decreased aflatoxin production at the concentration of 100 μg/mL via downregulation of *aflD*, *aflK*, and *aflQ* genes, while Compound **5** downregulated only the expression of the *aflS* gene. The potent inhibition of Compound **1** was related to downregulation of *aflK* gene responsible for VERB synthase expression to form versicolorin B, a key intermediate in aflatoxin biosynthesis. Taken together, Compound **1** can be developed as an antifungal and antiaflatoxigenic agent to control *A. flavus* and aflatoxin contamination in crop plants and stored products.

## 3. Materials and Methods

### 3.1. Chemicals 

The following compounds (26 compounds) were all purchased from Sigma-Aldrich Co. (St. Louise, MO, USA). Tested coumarins were 8-acetyl-7-hydroxycoumarin, 3-acetyl-6-bromocoumarin, 6-bromo-3-cyano-4-methyl-coumarin, 4-(bromomethyl)-6,7-dimethoxycoumarin, 3-cyano-7-hydroxy-4-methylcoumarin, 3-cyano-4,6-dimethyl-coumarin, coumarin, 4,6-dichloro-3-formylcoumarin, 6,7-dimethoxy-4-methylcoumarin, 5,7-dimethoxycoumarin, 7-ethoxy-4-methylcoumarin, 7-ethoxycoumarin, 4-hydroxy-6,7-dimethyl-coumarin, 7-methoxy-4-methylcoumarin, 6-methoxy-4-methylcoumarin, 7-methoxycoumarin, 7-hydroxy-6-methoxycoumarin, 4-hydroxy-7-methoxy-3-phenyl-coumarin, 6-methoxycoumarin, 5,6,7-trimethoxycoumarin, 6,7-dimethoxycoumarin, 6-methoxy-[7,8-(1-methoxy)methylenedioxy]coumarin, 6,7-(1-methoxy)methylenedioxy coumarin, 6,7,8-trimethoxycoumarin, 7-hydroxycoumarin, and dihydrocoumarin. In addition to these coumarin derivatives, some benzene-fused compounds were also tested for the evaluation of fungal and antiaflatoxigenic activities. 2,3-Dihydrobenzofuran, indole, 1-methyl indole, 2-methyl indole, 3-methyl indole, and 2-phenyl indole were also purchased from Sigma-Aldrich Co. (Figure 1).

### 3.2. Microorganisms and Preparation of the Spore Solution

*Aspergillus flavus* ATCC 22546 was purchased from the American Type Culture Collection (ATCC, Manassas, VA, USA) and was grown on malt extract agar (MEA: Difco Laboratories, Sparks, MD, USA). This isolate during the development generated aflatoxin B1 and B2, but not for G1 and G2 [23]. It was grown on MEA medium at 30 °C for 5 days until fungal spores were formed. After spore formation, they were collected from slants by shaking under 0.05% (*v*/*v*) Tween 80 and finally stored at −70 °C in a 20% glycerol solution (*v*/*v*).

### 3.3. Aflatoxin Analysis Using an HPLC-FLD

Fungal spore suspension adjusted to 10^6^ population was inoculated to the liquid culture media consisting of potato dextrose broth (25 mL) (PDB, Difco Laboratories). All tested compounds were spiked to the corresponding liquid media with a serial basis, and the culture was incubated at 25 °C for 5 days under shaking conditions. All experiments were triplicates for each concentration of the tested compound.

Following liquid medium cultivation for 5 days, the fungal growth was measured using filter paper to weigh the mycelial and sclerotial residues with overnight dryness in a dry oven. Separately, the mycelia from each treatment were subjected to the extraction procedure using an ultrasonic cleaner, and analyses of aflatoxin B (AFB) and G (AFG) was undertaken using an HPLC-FLD [20]. The average of the three replicates were calculated with standard deviation, and the data were compared with a control using one-way ANOVA at a *p* < 0.05 significance level [33]. 

### 3.4. Real-Time qPCR (RT-qPCR) after Isolation of Total RNA 

RT-qPCR was employed to understand the mode of the inhibitory effect on fungal growth and aflatoxin production. Fungal mycelia in liquid media were carefully collected and total RNA was extracted using the QIAzol Lysis reagent supplied by QIAGEN Inc. (Dusseldorf, Germany) after grinding to a fine powder under an appropriate amount of liquid nitrogen. Total RNAs extracted from the treated fungi were quantified by a μDrop™ Plate (Thermo Fisher Scientific Inc., Waltham, MA, USA), and the extracted RNAs were qualitatively checked using 1% agarose gel with ethidium bromide. Complementary DNA (cDNA) for extracted RNAs (2 μg) was synthesized using Maxima First Strand cDNA Synthesis Kit (Thermo Fisher Scientific Inc., Waltham, MA, USA).

RT-qPCR was undertaken by a Rotor-Gene SYBR Green PCR Kit (QIAGEN Inc.) with an proper amount of cDNA (100 ng). Primers for genes such as *yap*, *aflR*, *aflS*, *aflK*, *aflD*, *aflQ*, and *18S rRNA* were synthesized by Genotech (Daejeon, Korea), and they were used to understand the relationship between aflatoxin biosynthesis and the active compound [20]. Forty cycles of thermal cycling parameters were performed for amplification as follows: denaturation at 95 °C for 30 s, annealing at 60 °C for 20 s, and elongation at 72 °C for 30 s, followed by an additional step at 95 °C for 5 min. RT-qPCR was done triplicates for each treatment. Significant differences in gene expression were calculated using double delta Ct methods [34]. Data were standardized with *18S rRNA*, and gene expressions between the treatment and controls were compared using Prism 6 software (GraphPad, San Diego, CA, USA). Statistically significant differences between experimental groups were analyzed by a Student’s *t*-test (*p* < 0.05).

## Figures and Tables

**Table 1:** Mycelial growth of *Aspergillus flavus* treated with various coumarins.

Compounds	Treated Concentration (μg/mL)	Mycelial Growth (mg)
Thiabendazole (Positive control)	10	1.2 ± 2.1 (1.0%)
	5	6.3 ± 10.1 (5.0%)
	1	96.3 ± 2.4 (77.0%)
4-Hydroxy-6,7-dimethylcoumarin	1000	0.0 ± 0.00 (0%)
	100	22.5 ± 3.7 (18.0%)
	10	73.9 ± 17.4 (59.1%)
4-Hydroxy-7-methoxy-3-phenylcoumarin	1000	0.0 ± 0.00 (0%)
	100	41.1 ± 27.1 (32.8%)
	10	61.9 ± 14.1 (49.5%)
6,7-Dimethoxycoumarin	1000	0.0 ± 0.00 (0%)
	100	63.0 ± 44.3 (50.4%)
	10	93.7 ± 8.3 (74.9%)
2,3-dihydrobenzofuran	1000	0.0 ± 0.00 (0%)
	100	152.3.0 ± 45.1 (124%)
4-(Bromomethyl)-6,7-dimethoxycoumarin	1000	0.0 ± 0.00 (0%)
	100	46.4 ± 3.7 (37.1%)
	10	120.9 ± 18.5 (96.7%)

Mycelial growth for the negative control was 124.0 ± 23.0 mg obtained from three experiments.

**Table 2:** Inhibitory effects of coumarins on aflatoxin production in *Aspergillus flavus.*

Compounds	Treated Conc. (μg/mL)	Aflatoxin Production (ng/mL)			
		Aflatoxin B1	Aflatoxin B2	Aflatoxin G1	Aflatoxin G2
Control	-	1928.9 ± 403.4 *^a^*	37.2 ± 6.3 *^a^*	184.3 ± 66.5 *^a^*	29.5 ± 5.3 *^a^*
Thiabendazole	5	ND *^,*b*^	ND *^b^*	64.4 ± 2.6 *^b^*	ND *^b^*
	1	−	−	239.2 ± 65.7 *^a^*	−
3-Acetyl-6-bromocoumarin	10	ND *^b^*	ND *^b^*	92.6 ± 25.7 *^b^*	ND *^b^*
	1	−	−	−	−
4-Hydroxy-6,7-dimethyl-coumarin	10	ND *^b^*	ND *^b^*	ND *^b^*	ND *^b^*
	1	−	ND *^b^*	−	ND *^b^*
4-Hydroxy-7-methoxy-3-phenyl-coumarin	100	ND *^b^*	ND *^b^*	ND *^b^*	ND *^b^*
	10	158.8 ± 25.6 *^c^*	ND *^b^*	192.6 ± 32.4 *^a^*	ND *^b^*
	1	1025.4 ± 329.9 *^d^*	19.3 ± 3.4 *^c^*	137.8 ± 39.0 *^a^*	5.3 ± 1.0 *^c^*
Dihydrocoumarin	1000	ND *^b^*	ND *^b^*	ND *^b^*	ND *^b^*
	10	1397.5 ± 675.9 *^a^*	28.8 ± 7.8 *^b^*	99.6 ± 62.0 *^b^*	ND *^b^*
	1	2517.9 ± 199.8 *^a^*	49.7 ± 36.5 *^a^*	164.6 ± 120.8 *^a^*	5.1 ± 1.7 *^c^*
2,3-Dihydrobenzofuran	1000	ND *^b^*	ND *^b^*	ND *^b^*	ND *^b^*
	100	ND *^b^*	ND *^b^*	ND *^b^*	ND *^b^*
	1	1140.0 ± 342.1 *^c^*	21.8 ± 5.1 *^c^*	123.3 ± 33.7 *^c^*	5.8 ± 2.6 *^c^*
4-(Bromomethyl)-6,7-dimehtoxycoumarin	1000	ND *^b^*	ND *^b^*	ND *^b^*	ND *^b^*
	100	ND *^b^*	ND *^b^*	ND *^b^*	ND *^b^*
	10	−	−	−	ND *^b^*

* ND: Not detectable; −: means more than 150% aflatoxin production in comparison to that of the control. Statistical analysis performed and different letters in the same column indicate significantly different from the control group (*p* < 0.05).
